# Data on gender contrasts in the risk of incident myocardial infarction by age. The Tromsø Study 1979–2012

**DOI:** 10.1016/j.dib.2017.07.001

**Published:** 2017-07-08

**Authors:** Grethe Albrektsen, Ivar Heuch, Maja-Lisa Løchen, Dag Steinar Thelle, Tom Wilsgaard, Inger Njølstad, Kaare Harald Bønaa

**Affiliations:** a Department of Public Health and Nursing, Faculty of Medicine and Health Science, NTNU - Norwegian University of Science and Technology, Trondheim, Norway; b Department of Mathematics, University of Bergen, Bergen, Norway; c Department of Community Medicine, Faculty of Health Sciences, UiT - The Arctic University of Norway, Tromsø, Norway; d Department of Biostatistics, Institute of Basic Medical Sciences, University of Oslo, Oslo, Norway; e Section for Epidemiology and Social Medicine, Sahlgrenska Academy, University of Gothenburg, Gothenburg, Sweden; f Clinic for Heart Disease, St. Olavs University Hospital, Trondheim, Norway

**Keywords:** Myocardial infarction, Gender, Age, Lipids, Blood pressure, Smoking, Relative risk

## Abstract

The data presented in this article relate to the research article entitled “Risk of incident myocardial infarction by gender: Interactions with serum lipids, blood pressure and smoking. The Tromsø Study 1979–2012” (Albrektsen et al., 2017) [1]. Data quantify the gender differences in the risk of myocardial infarction (MI) in terms of incidence rate ratios (IRR), in subgroups defined by serum lipids, blood pressure and smoking among persons aged 35–54 years, 55–74 years and 75–94 years, respectively. Data also describe the age- and gender-specific linear associations with the coronary heart disease (CHD) risk factors. IRRs for combined categories of age, gender and a CHD risk factor, with each category compared to the same reference group, are also shown. IRRs were calculated as estimates of relative risk in Poisson regression analyses of person-years at risk. Among 33,859 individuals at risk, a total of 622, 1308 and 816 were diagnosed with MI at ages 35–54, 55–74 and 75–94 years, respectively.

**Specifications Table**

**Table:** 

Subject area	*Medicine*
More specific subject area	*Coronary heart disease epidemiology*
Type of data	*Tables and Figures*
How data was acquired	*Poisson regression analyses of person-years at risk, with calculations based on information obtained from a large population-based prospective study*
Data format	*Analyzed*
Experimental factors	
Experimental features	
Data source location	*Tromsø, Norway*
Data accessibility	*The data are available with this article*

**Value of the data**

• Data can be used for identifying subgroups where the gender contrast in risk of incident MI is particularly high or low.
• Data can be used for evaluation of gender heterogeneity in the association with established CHD risk factors.
• Data can be used for comparing risk of incident MI between any subgroups defined by age, gender and a CHD risk factor.
• Data can be utilized for exploring issues that can improve knowledge on biological mechanisms underlying the gender contrast in the risk of CHD.
• At the community level, data can be utilized for development of gender-specific CHD risk preventive guidelines.

## 1 Data

**Fig. 1:**
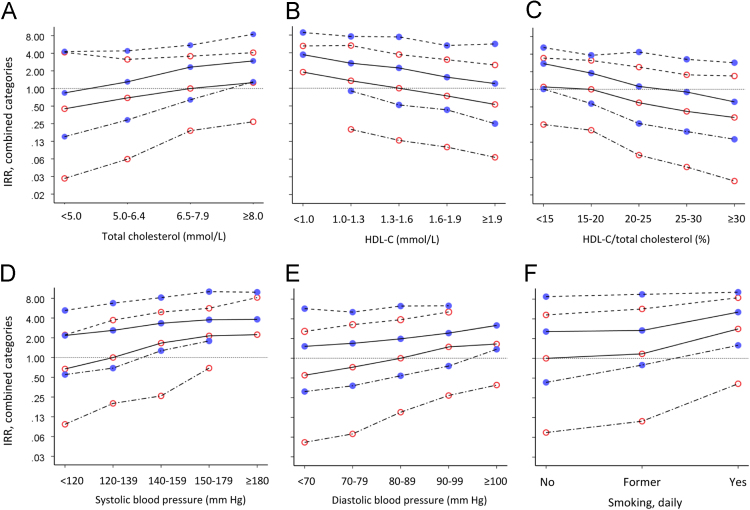
**Relative risk in combined categories of interacting factors**. Adjusted incidence rate ratio (IRR, on logarithmic scale) of myocardial infarction in combined categories of age (— . — 35-54 yr, _____ 55-74 yr, - - - - 75-94 yr), gender ( men,  women) and (A) total cholesterol, (B) HDL-C, (C) HDL-C in percent of total cholesterol, (D) systolic BP, (E) diastolic BP, (F) daily smoking. The horizontal reference line (IRR = 1.00) in each figure goes through the common reference group.

Data displayed in Table 1 are age- and gender-specific incidence rate ratios (IRR) of myocardial infarction (MI) with 95% confidence intervals (CI) for the linear associations with total cholesterol, high-density lipoprotein cholesterol (HDL-C), and HDL-C in percent of total cholesterol. Data shown in Table 2 are the age-specific IRR for gender (men vs. women) within categories defined by the lipid components. Table 3, Table 4 show corresponding numbers for the interaction between gender and systolic and diastolic blood pressure, and Table 5 shows IRR for the interaction between gender and daily smoking. The number of MI-diagnoses within each subgroup defined by age, gender and a CHD risk factor, is also shown. Fig. 1A-F show IRR values from analyses of combined categories of age (35–54, 55–74 and 75–94 years), gender and each CHD risk factor, with each subgroup compared to the same reference group. The data are original and have not been published elsewhere.

**Table 1:** Age- and gender-specific incidence rate ratio of myocardial infarction for linear association with serum lipids. The Tromsø Study 1979–2012.

	35–54 years	55–74 years	75–94 years	
	IRR (95% CI)^a^	IRR (95% CI)^a^	IRR (95% CI)^a^	*p*, interaction^b^
TC (per 1.50 mmol/L)^c^^,^^d^				
– Men	2.02 (1.82–2.23)	1.54 (1.43–1.67)	1.21 (1.07–1.38)	<0.001
– Women	2.10 (1.64–2.70)	1.38 (1.21–1.56)	1.06 (0.95–1.20)	<0.001
*p*, interaction^e^	0.75	0.12	0.13	
HDL-C (per 0.30 mmol/L)^c^^,^^d^				
– Men	0.72 (0.66–0.79)	0.76 (0.72–0.81)	0.91 (0.84–0.99)	<0.001
– Women	0.76 (0.63–0.92)	0.73 (0.67–0.80)	0.82 (0.76–0.88)	0.27
*p*, interaction^e^	0.62	0.44	0.062	
HDL-C/TC (per 5%)				
– Men	0.60 (0.55–0.64)	0.68 (0.64–0.72)	0.89 (0.82–0.96)	<0.001
– Women	0.57 (0.48–0.68)	0.71 (0.65–0.78)	0.81 (0.75–0.88)	<0.001
*p*, interaction^e^	0.66	0.42	0.12	

IRR, incidence rate ratio; CI, confidence interval; TC, total cholesterol; HDL-C, high-density lipoprotein cholesterol.

a IRR for linear trend through ordered lipid categories (see Table 2), adjusted for age (1-year interval, categorical), birth-cohort (5-year categories), diastolic blood pressure (linear trend) and daily smoking.

b Likelihood ratio test for linear interaction (age*lipid component) for men and women.

c Additional adjustment for HDL-C or total cholesterol.

d To convert mmol/L to mg/dL, divide by 0.0259 (or multiply with 38.61004).

e Likelihood ratio test for linear interaction (gender*lipid component) within age groups.

**Table 2:** Age-specific incidence rate ratio of myocardial infarction for gender within serum lipid categories. The Tromsø Study 1979–2012.

	35–54 years			55–74 years			75–94 years		
	No. of MI		IRR (95% CI)^a^	No. of MI		IRR (95% CI)^a^	No. of MI		IRR (95% CI)^a^
	M	W	Men vs. women	M	W	Men vs. women	M	W	Men vs. women
TC (mmol/L)^b^^,^^c^									
<5.0	34	7	4.73 (2.09–10.7)	60	17	1.87 (1.09–3.21)	57	31	1.13 (0.72–1.76)
5.0–6.4	165	27	4.21 (2.79–6.36)	335	107	1.85 (1.48–2.31)	172	116	1.55 (1.21–1.98)
6.5–7.9	223	35	3.22 (2.24–4.62)	418	152	2.27 (1.87–2.75)	124	184	1.68 (1.31–2.14)
≥8.0	120	11	4.61 (2.54–8.39)	146	73	2.28 (1.71–3.03)	38	94	2.23 (1.52–3.29)
*p*, interaction^d^			0.62			0.49			0.15
HDL-C (mmol/L)^b^^,^^c^									
<1.00	107	3	–e	150	26	1.96 (1.29–2.98)	48	28	1.44 (0.90–2.30)
1.0–1.29	243	21	4.37 (2.91–6.57)	351	81	1.98 (1.55–2.53)	132	104	1.35 (1.03–1.77)
1.3–1.59	126	26	3.58 (2.34–5.46)	282	107	2.21 (1.77–2.77)	116	115	1.72 (1.31–2.25)
1.6–1.89	48	18	3.92 (2.28–6.75)	114	76	2.09 (1.56–2.80)	52	88	1.60 (1.12–2.28)
≥1.90	18	12	3.33 (1.60–6.92)	62	59	2.30 (1.61–3.30)	43	90	2.16 (1.48–3.14)
*p*, interaction^d^			0.89			0.94			0.33
HDL-C/TC (%)									
<15	161	9	4.15 (2.12–8.13)	195	38	2.48 (1.75–3.52)	48	48	1.57 (1.04–2.36)
15–19.9	211	26	2.91 (1.94–4.38)	365	108	1.89 (1.52–2.35)	101	122	1.25 (0.95–1.65)
20–24.9	94	18	3.49 (2.10–5.78)	217	94	1.92 (1.50–2.45)	121	124	1.89 (1.45–2.45)
25–29.9	45	13	4.03 (2.17–7.47)	114	55	2.13 (1.54–2.94)	65	65	1.96 (1.38–2.79)
≥30	31	14	4.84 (2.57–9.12)	68	54	1.86 (1.30–2.66)	56	66	1.74 (1.21–2.51)
*p*, interaction^d^			0.70			0.69			0.17

MI, myocardial infarction; IRR, incidence rate ratio; CI, confidence interval; M, men; W, women; TC, total cholesterol; HDL-C, high-density lipoprotein cholesterol.

a Adjusted for age (1-year interval, categorical), birth-cohort (5-year categories), diastolic blood pressure (linear trend) and daily smoking.

b Additional adjustment for HDL-C or total cholesterol.

c To convert mmol/L to mg/dL, divide by 0.0259 (or multiply with 38.61004).

d Likelihood ratio test for categorical interaction (gender*lipid component) within age groups.

e Two first categories collapsed.

**Table 3:** Age- and gender-specific incidence rate ratio of myocardial infarction for linear association with blood pressure. The Tromsø study 1979–2012.

	35–54 years	55–74 years	75–94 years	
	IRR (95% CI)^a^	IRR (95% CI)^a^	IRR (95% CI)^a^	*p*, interaction^b^
SBP (per 20 mmHg)				
– Men	1.53 (1.38–1.70)	1.21 (1.14–1.29)	1.16 (1.06–1.27)	0.002
– Women	1.70 (1.36–2.13)	1.41 (1.29–1.55)	1.29 (1.18–1.41)	0.39
*p*, interaction^c^	0.40	0.007	0.10	
DBP (per 10 mmHg)				
– Men	1.45 (1.33–1.58)	1.20 (1.13–1.28)	1.07 (0.97–1.18)	<0.001
– Women	1.74 (1.42–2.13)	1.34 (1.22–1.48)	1.24 (1.13–1.37)	0.039
*p*, interaction^c^	0.095	0.045	0.038	

IRR, incidence rate ratio; CI, confidence interval; SBP, systolic blood pressure; DBP, diastolic blood pressure.

a IRR for linear trend through ordered blood pressure categories (see Table 4), adjusted for age (1-year interval, categorical), birth-cohort (5-year categories), HDL-C in percent of total cholesterol (linear trend), and daily smoking.

b Likelihood ratio test for linear interaction (age*BP) for men and women.

c Likelihood ratio test for linear interaction (gender*BP) within age groups.

**Table 4:** Age-specific incidence rate ratio of myocardial infarction for gender within blood pressure categories. The Tromsø Study 1979–2012.

	35–54 years			55–74 years			75–94 years		
	No. of MI		IRR (95% CI)^a^	No. of MI		IRR (95% CI)^a^	No. of MI		IRR (95% CI)^a^
	M	W	Men vs. women	M	W	Men vs. women	M	W	Men vs. women
SBP (mmHg)									
<120	54	18	4.67 (2.73–8.01)	75	30	3.20 (2.09–4.89)	16	8	2.52 (1.08–5.90)
120–139	250	39	3.02 (2.15–4.26)	345	88	2.53 (2.00–3.20)	77	53	1.91 (1.34–2.72)
140–159	188	14	4.45 (2.58–7.68)	336	119	1.95 (1.58–2.42)	132	114	1.75 (1.35–2.27)
160–179	41	5	2.31 (1.13–4.71)	153	77	1.73 (1.31–2.28)	108	120	1.91 (1.46–2.51)
≥180	9	4	–b	50	35	1.70 (1.10–2.62)	58	130	1.30 (0.94–1.79)
*p*, interaction^c^			0.28			0.047			0.28
DBP (mmHg)									
<70	41	10	5.33 (2.66–10.7)	50	37	2.75 (1.80–4.21)	48	44	2.34 (1.55–3.54)
70–79	130	19	4.69 (2.89–7.62)	212	84	2.30 (1.78–2.96)	94	93	1.63 (1.21–2.18)
80–89	196	28	3.21 (2.15–4.78)	342	121	1.95 (1.58–2.40)	150	157	1.69 (1.33–2.14)
90–99	109	17	2.58 (1.54–4.31)	237	78	1.62 (1.25–2.10)	90	126	1.36 (1.04–1.80)
≥100	66	6	3.03 (1.31–7.01)	118	29	1.91 (1.27–2.87)	9	5	–b
*p*, interaction^c^			0.34			0.20			0.18

MI, myocardial infarction; IRR, incidence rate ratio; CI, confidence interval; M, men; W, women; SBP, systolic blood pressure; DBP, diastolic blood pressure.

a Adjusted for age (1-year interval, categorical), birth-cohort (5-year categories), HDL-C in percent of total cholesterol (linear trend), and daily smoking.

b Two last categories collapsed.

c Likelihood ratio test for categorical interaction (gender*BP) within age groups.

**Table 5:** Age-specific incidence rate ratio of myocardial infarction for gender in smokers and non-smokers, and age- and gender-specific associations with smoking. The Tromsø Study 1979–2012.

	35–54 years			55–74 years			75–94 years		
	No. of MI		IRR (95% CI)^a^	No. of MI		IRR (95% CI)^a^	No. of MI		IRR (95% CI)^a^
	M	W	Men vs. women	M	W	Men vs. women	M	W	Men vs. women
Smoking, daily									
No	60	8	4.57 (2.17–9.60)	143	88	2.51 (1.92–3.28)	62	237	1.95 (1.47–2.58)
Former (F)	108	8	5.27 (2.56–10.9)	302	65	2.21 (1.67–2.90)	240	100	1.75 (1.38–2.21)
Yes	374	64	3.25 (2.48–4.27)	514	196	1.76 (1.48–2.08)	89	88	1.23 (0.91–1.65)
IRR, F vs. No									
– Men			1.80 (1.31–2.48)			1.04 (0.85–1.27)			1.12 (0.81–1.55)
– Women			1.56 (0.59–4.16)			1.18 (0.86–1.63)			1.20 (0.95–1.52)
IRR, Yes vs. No									
– Men			3.68 (2.79–4.85)			1.91 (1.59–2.31)			1.08 (0.82–1.43)
– Women			5.17 (2.47–10.8)			2.74 (2.13–3.52)			1.78 (1.39–2.28)
*p*, interaction^b^			0.34			0.057			0.067

MI, myocardial infarction; IRR, incidence rate ratio; CI, confidence interval; M, men; W, women.

a Adjusted for age (1-year interval, categorical), birth-cohort (5-year categories), HDL-C in percent of total cholesterol (linear trend) and diastolic blood pressure (linear trend).

b Likelihood ratio test for categorical interaction (gender*smoking) within age groups.

## 2 Experimental design, materials and methods

### 2.1 Population at risk

Data displayed are calculated on the basis of information from the Tromsø Study in Norway [1], [2]. Individual risk factor levels were obtained through questionnaires, blood samples and physical examinations in five repeated surveys in the calendar period 1979–2008. CHD risk factor levels in men and women at start of follow-up are given elsewhere [3]. Dates of MI-diagnoses, emigration and deaths in the period 1979–2012 were obtained from local and national registers. Among 33,859 individuals at risk (51% women), a total of 622 (80 women), 1308 (349 women) and 816 (425 women) had an MI at ages 35–54, 55–74 and 75–94 years, respectively.

### 2.2 Statistical analysis

The data are obtained from Poisson regression analyses of person-years at risk, with IRR of incident MI calculated as estimates of relative risk [4], [5]. Information from all repeated surveys was utilized [1]. The data shown in Table 1, Table 2, Table 3, Table 4, Table 5 are calculated on the basis of two-way interaction models (between gender and a CHD risk factor) in separate analyses of persons aged 35–54, 55–74 and 75–94 years (corresponding to a three-way interaction model). Within each broad age group, IRRs were adjusted for age in 1-year categories. The data displayed in Table 1, Table 2, Table 3, Table 4, Table 5 quantify subgroup-specific associations with each single risk factor, but provide no information on whether a high-risk group among young people encloses, or possibly crosses the risk level of any older subgroup.

Data displayed in Fig. 1A–F are obtained from analyses of a single variable representing combined categories of age (35–54, 55–74 and 75–94 years), gender and a CHD risk factor (a unique value assigned to each possible value combination). A subgroup in middle-aged women with sufficient number of MI cases, as close as possible to normal-range or unexposed for the CHD risk factor considered, was used as common reference group when calculating the IRRs, and the risk estimates for all subgroups can be compared directly. The internal order of the IRRs will also reflect the rank of absolute risks. To ensure that persons in one particular broad age group were compared with persons exactly 20 years older or younger, additional indicator variables for age were included in the model (1-year categories, original age variable recoded 1–20 within each broad age group). The data presented in Fig. 1A–F provide information on whether a high-risk group in young people encloses the risk level of any older subgroup, but do not quantify the association with each single risk factor.

The regression models used for generating the data are an extension of the two-way interaction models applied in the original research paper [1], to three-way-interaction models used for evaluation of homogeneity across age-groups.
